# The association between emotional arousal and color lightness of facial digital expressions of social robots

**DOI:** 10.3389/fpsyg.2026.1677997

**Published:** 2026-02-06

**Authors:** Xu Chengxing, Fu Ying, Ren Aonini, Wang Qiong

**Affiliations:** 1 Nanjing University of Posts and Telecommunications, School of Digital Media and Design Art, Nanjing, China; 2 Changzhou University, School of Art and Design, Changzhou, China

**Keywords:** social robots, color lightness, human-computer interaction, emoticons, emotional arousal, visual communication, psychology

## Abstract

In human–robot social interactions, a robot’s facial expressions serve as a crucial channel for conveying emotions and fostering trust. However, current social robots predominantly employ emoji-like icons and simulated muscle movements for anthropomorphic ex-pression, and research on facial color–emotion relationships tends to adjust hue, saturation, and lightness simultaneously—overlooking how color lightness alone influences emotional intensity. This study explores the specific link between emotion type and color lightness in social robots’ digital facial expressions to enhance affective communication in human–computer interaction. In our experiment, participants viewed robot faces dis-playing the same emotion at varying lightness levels and rated perceived arousal on a 1–5 scale. The results confirm that manipulating color lightness significantly modulates emotional arousal. Overall, these findings suggest that adaptive lightness adjustments in robot facial colors can yield more realistic and intuitive affective interactions between humans and machines.

## 1 Introduction

In daily social interactions, individuals rely heavily on facial cues to infer others’ emotional states, guiding judgments about the surrounding atmosphere and underlying intentions. Facial emotional expression serves as a vital non-verbal channel for conveying personal intent and fostering rapport, both in human–human and human–robot contexts (Van Kleef and Côté, 2022). Effective emotional arousal enhances the vividness and clarity of visual emotion displays, thereby strengthening the observer’s affective engagement (Mourao-Miranda et al., 2003). During ongoing human–machine interactions, visual features become the primary modality through which users seek and perceive emotional information. Accordingly, social robots that exhibit clearly recognizable emotional ex-pressions positively influence user comfort, trust, and engagement. Moreover, robots endowed with perceivable affective characteristics can function as mediators, aiding humans in understanding and responding to others’ emotions (Spezialetti et al., 2020; Mavridis, 2015). Simulating physiological signals, such as skin tone or vascular changes via color adjustments on the robot’s face directly enhances the authenticity of emotional transmission (Giard and Guitton, 2010; Cai et al., 2023). At present, the main approach for social robots (such as Pepper and NAO) to achieve social interaction goals is to regulate color and expression changes through the LED panels on their faces. By doing so, these robots can provide users with “facial cues,” thereby facilitating the transmission of emotions. In the face of the complex and diverse characteristics of human emotions, the process of robots processing emotional data requires more accurate and rapid discrimination to achieve effective social interaction with humans. According to Russell’s (1980) circular model, human emotions can be divided into a two-dimensional space of “high/low valence” and “high/low arousal,” and all emotions can be located in this space, with different emotions and arousal levels having certain differences. Plutchik’s (1980) emotion wheel theory indicates that there are basic emotions, and each emotion has an “intensity hierarchy.” In the process of choosing facial colors, the rationality of the initial hue se-lection is particularly important. Therefore, the facial color of robots is a perceptual feature that can be directly obtained. The change of color relies on the numerical adjustment among hue, saturation and lightness. Previous human-robot interaction studies usually changed hue and saturation simultaneously; few studies kept hue/saturation constant to separately study lightness. Therefore, the connection between lightness and emotional arousal is effective and direct, and the independent influence of lightness on color needs to be studied.

Social robots rely on facial cues to communicate affect and guide social responses. Prior work has mapped color–emotion links mostly by co-varying hue, saturation, and lightness, which obscures the independent role of lightness. This gap is consequential for resource-constrained robots where lightness modulation can operate as a device-independent, low-bandwidth channel for affective display at conversational distances. We isolate lightness while holding hue and saturation constant and ask whether graded lightness of emoji-style facial displays systematically modulates perceived arousal across basic emotions. We provide behavioral evidence that lightness alone shapes arousal judgments (Experiment 1), and a design implication for timing alignment in dynamic displays (Experiment 2). Together these findings position lightness as a deployable coding channel for affective HRI and clarify when high vs. low lightness best supports specific emotions.

## 2 Literature review

While previous studies have explored links between hue and emotional states, such as red indicating anger and blue suggesting calm, most have co-varied hue, saturation, and lightness, which obscures the independent role of lightness in emotional expression. In human–robot interaction, variations in color lightness and saturation both affect perceived emotion, yet their relationship in the HSL space requires nuanced treatment: near the extremes of lightness (approaching pure black/white), effective chroma collapses (a chroma-dissolution effect), whereas within moderate lightness ranges, maintaining comparable perceptual intensity may involve coordinated adjustments to saturation patterns that remain hue and emotion dependent. Accordingly, within human–robot interaction, fluctuations in lightness are not merely cosmetic; they can alter the clarity and intensity of emotional signals. On this view, changes in color lightness directly shape emotional communication, while its perceptual coupling with saturation (especially at extreme lightness) must be considered when interpreting HSL manipulations (Jonauskaite et al., 2019; Valdez and Mehrabian, 1994; Corney et al., 2009). For instance, human anger is physiologically associated with increased blood flow and can be depicted with red for excited/angry expressions. Intense emotions (e.g., anger, excitement) co-occur with peripheral physiological changes that manifest on the face as shifts in color and lightness. Peromaa and Olkkonen (2019) argue that such states activate the autonomic nervous system, leading to vasodilation-widening of blood vessels-and increased facial blood flow, producing subtle but detectable reddening or flushing. These changes vary with individual factors (e.g., skin tone, vascular reactivity). Using colorimetric analysis, they measured variations in skin lightness and hue, providing empirical support for the long-noted idea that emotions “show on one’s face.” The implications span psychology, behavioral science, computer vision, and affective computing, where recognizing emotion from facial cues is increasingly important. Overall, adjusting color values (saturation, lightness, hue) can promote emotional arousal, while color selection modulates facial emotional valence (Kuniecki et al., 2015) and facilitates unconscious emotional activation and regulation (Lapate et al., 2014). As a key factor in arousal, color influences both recognition of facial expressions and valence judgments. However, the specific relationship between lightness changes on robotic faces and particular emotional expressions remains under-tested.

**Figure 1:**
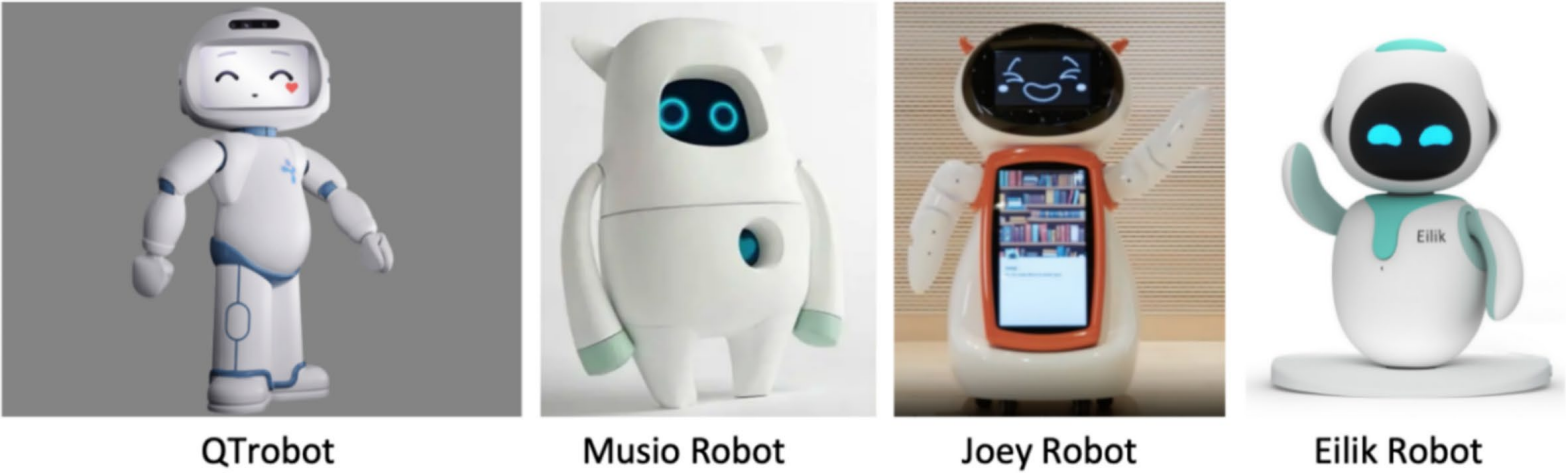
Facial emotion expression styles of robots in the market.

At present, commercially available social robots typically employ anthropomorphic digital screens or mechanically actuated bionic faces; emotional expressions are conveyed via external contours and emoji-style graphics on LED displays. As illustrated in Figure 1, robots render digital facial expressions by manipulating key shape elements (eyes, mouth) with eyebrows as auxiliary cues to mimic human feature changes across emotions. Compared with musculature-level mimicry, preset emoji’s on robot faces can enhance emotional transmission and user engagement (Riordan, 2017). In practice, however, dynamic color control often defaults to simple hue shifts (e.g., red to blue), which can be monotonic and insufficient for distinguishing high- vs. low-arousal states; overlooking lightness may reduce perceived intensity and nuance (Thanh Huyen et al., 2025; Chuah and Yu, 2021). By contrast, lightness dynamics play a central role: higher lightness is often associated with more positive impressions, whereas lower lightness conveys negative impressions. Modulating facial lightness can increase the authenticity and recognizability of robot expressions and strengthen users’ emotional connection during service/interaction. Current products and studies frequently link fixed colors with emotions, yet in-depth tests of how lightness, with hue and saturation held constant, modulates facial emotional arousal remain limited.

Guided by Russell’s (1980) circumplex model of affect (valence × arousal), we focus on lightness as the manipulated dimension while holding hue and saturation constant. Rather than toggling discrete hues (e.g., red vs. blue), we consider graded lightness changes—for example, the transition from bright red to near-black as anger intensifies (Casimir and Schnegg, 2002)—by decomposing these dynamics into static emoji displays for precise ratings. In line with prior work showing that facial lightness can modulate perceived authenticity and intensity (Luo and Dzhelyova, 2020), we examine whether graded lightness alone systematically modulates perceived arousal across basic emotions (joy, anger, fear, sadness). Participants rate perceived arousal for each lightness level on a 1–5 scale (L0 = 100% … L10 = 1%), which isolates lightness from hue/saturation and enables precise mapping of lightness–arousal correspondences. Ultimately, we assess whether observers form consistent lightness–emotion associations when interacting with social-robot faces, addressing an open gap in affective HRI.

## 3 Experiment 1

### 3.1 Materials and methods

A total of 47 participants (mean age = 21 years; 53% female), all of whom were university students, took part in this study. We targeted young adults to minimize known age-related variability in color perception and to reduce heterogeneity in affective judgments under controlled lab conditions (Bosten, 2022). Gender was recorded for descriptive completeness; the study was not powered to test gender effects and did not include gender as a factor. Participation was anonymous, and all volunteers had normal (or corrected-to-normal) vision and normal color perception. Recruitment was conducted in person, and each individual completed the study only once. No compensation (monetary or course credit) was provided for participation. Data collection was finalized before any analyses were performed; we report all data, procedures, and variables in full. The required sample size was determined *a priori* via power analysis, targeting 80% power to detect an effect of *d* = 0.50 at *α* = 0.05. The final sample slightly exceeded this target, confirming that our study was adequately powered for its aims.

Prior to testing, all participants provided written informed consent that covered publication of aggregated, anonymized results. Only minimal demographics (age, gender) were collected; no identifiers were stored with the responses. Data were stored on secure institutional servers with access restricted to the research team. The study complied with the Declaration of Helsinki and received approval from the Ethics Committee of Nanjing University of Posts and Telecommunications (NUPT EC). In line with the approval, individual-level raw data are not publicly released; de-identified summaries and analysis materials are available upon reasonable request subject to ethics constraints.

### 3.2 Stimuli and apparatus

**Figure 2:**
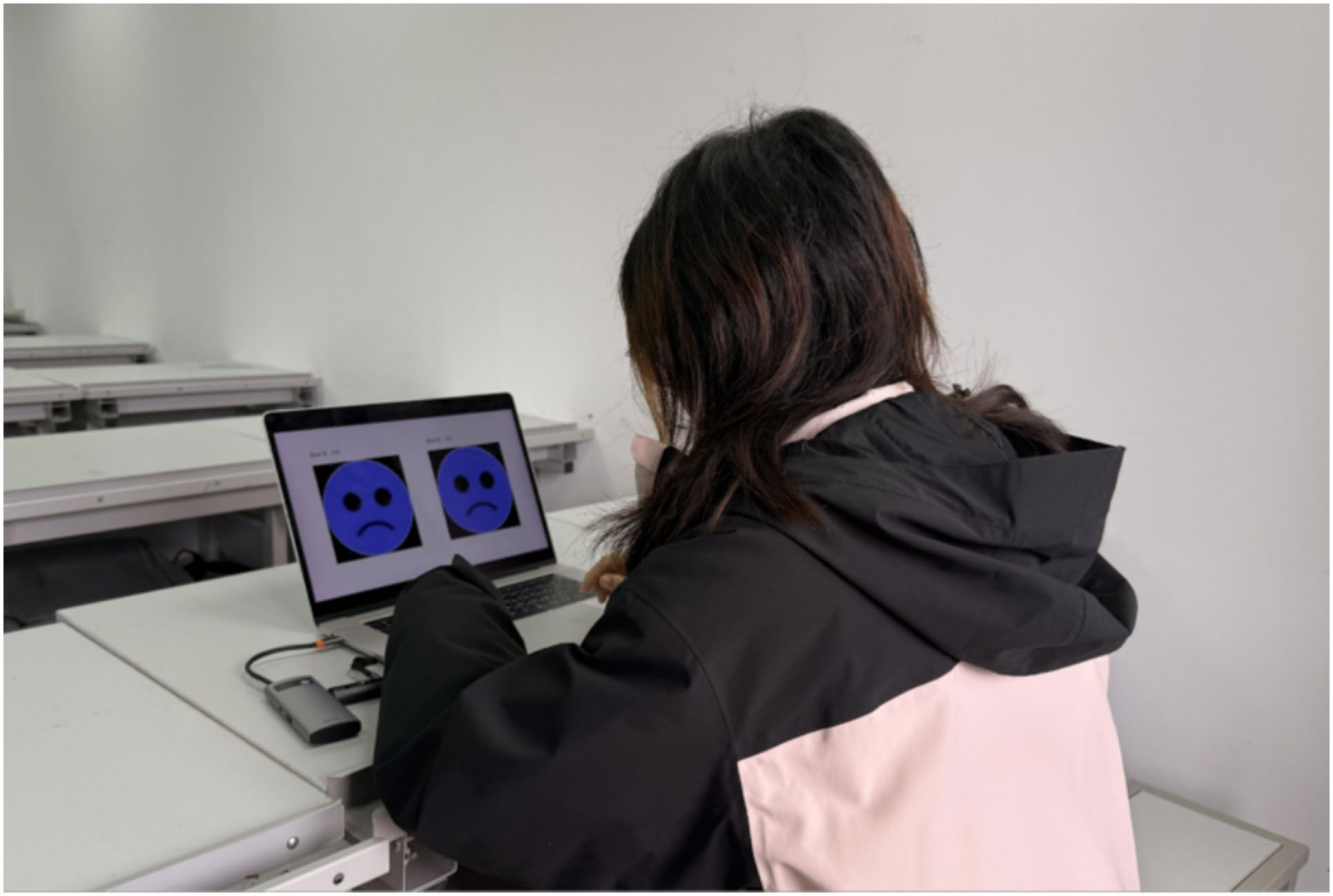
Experimental setup (illustrative).

The generation of experimental materials followed the dynamic facial synthesis methods of Nakajima et al. (2017), adapted here by replacing facial muscle movements with emoji color lightness changes. Basic emoji shapes were selected in accordance with Liao et al. (2018) cross-cultural emoticon taxonomy. Participants were presented with a set of five emotions (anger, happy, sad, fear, and neutral), chosen because these categories encompass the core set of human emotions identified in facial expression research (Jack et al., 2014; Izard, 2007). In all experiments, the same model of display (MacBook) was employed, along with a uniform neutral background (mid-gray or white under stable indoor lighting). The indoor lighting conditions were maintained at a stable level. Each participant maintained a consistent viewing distance of 35 cm from the display. Although the absolute lightness values (cd/m^2^) were not documented, the relative order of lightness levels remained consistent across all participants. Each trial adhered to the same sequence: (1) fixation point; (2) stimulus; (3) rating. This description generalizes the scenario presentation approach employed in all the trials of this research. See Figure 2 for an illustrative view of the setup.

**Figure 3:**
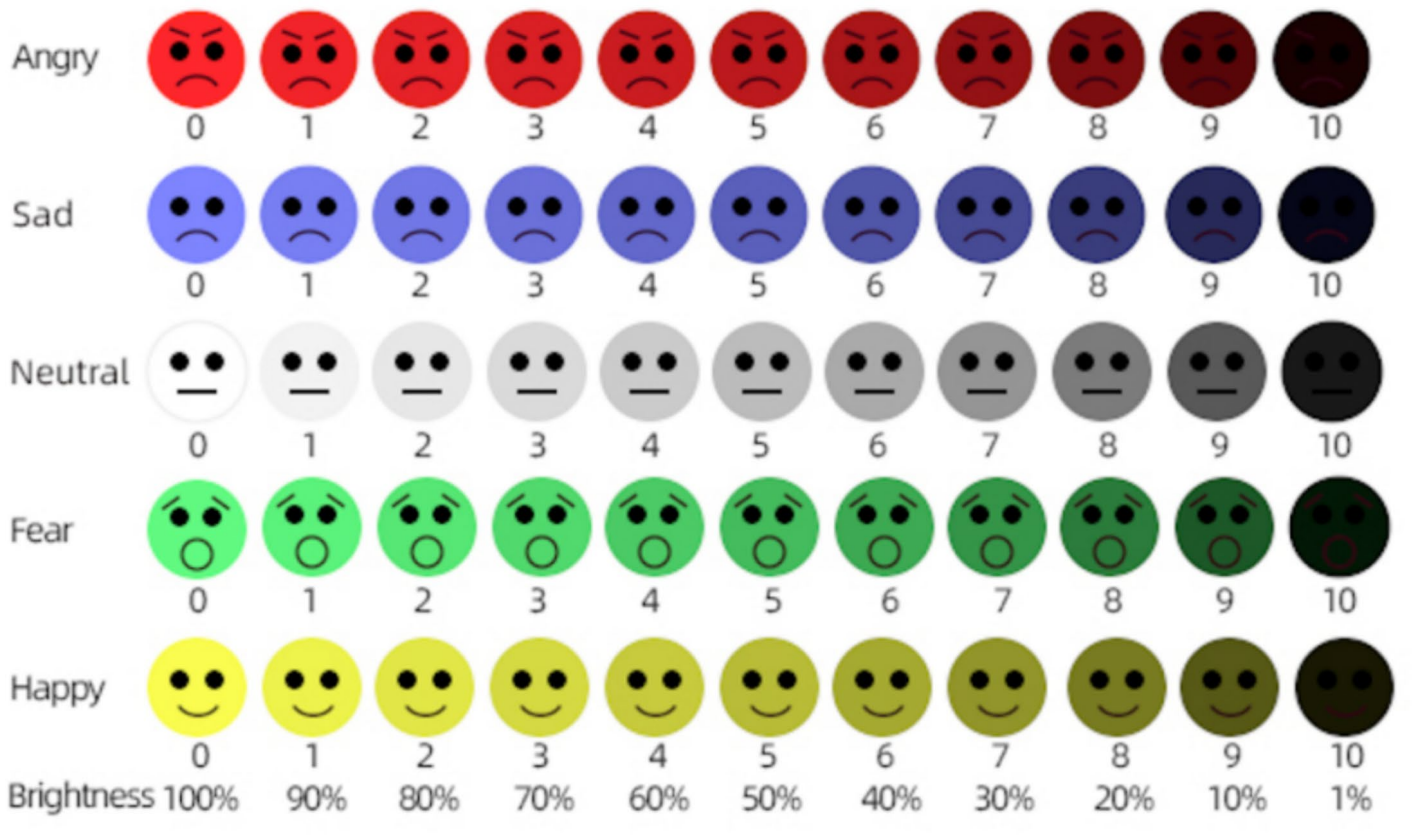
Stimulus set: basic emotions by lightness levels (hue and saturation fixed).

The experiment was designed based on the emotional color associations in Plutchik’s Emotion Wheel and the lightness values defined in the Munsell Color System. Lightness samples were created at 10-unit intervals. The colors corresponding to the target emotions in Plutchik’s Emotion Wheel were used as the initial emotional colors and assigned the highest lightness value, with subsequent samples decreasing in lightness at regular intervals. For the neutral emotion, light gray was selected as the most appropriate color (Liao et al., 2022). During the experiment, hue and saturation were held constant, and only the lightness component was manipulated. This ensured that differences in lightness were both distinct and easily perceived by participants. Participants rated perceived emotional intensity for each lightness level on a 1–5 Likert scale. To ensure minimal color recognizability, the lowest lightness value was set at 1%, as shown in Figure 3. In addition to collecting basic demographic information, the relationship between facial emotion perception and color lightness was assessed using a 5-point Likert scale. This figure illustrates the discrete stimuli used in Experiment1 (5emotions × 11lightness levels). It is not an inferential relationship plot; all statistical results are reported in Section 3.4 (Results). Mapping: L0 = 100% … L10 = 1% (higher labels indicate lower lightness). We use lightness (with hue and saturation held constant) to describe the achromatic dimension of the stimuli.

### 3.3 Procedures

The experiment was carried out in a quiet room. At the start of each session, participants completed basic demographic questions (e.g., gender, age). Participants were informed that, within each block, the displays represented the same basic emotion shown at different lightness levels. They then rated the perceived emotional intensity on a 5-point Likert scale. The experiment incorporated four basic emotions (anger, happiness, sadness, fear) and one neutral emotion. For each emotion, there were 11 distinct levels of color lightness. The lightness scale was anchored at the emotion color in Plutchik’s wheel (highest lightness), labeled L0, and then decreased sequentially to L10. In total, participants completed 55 trials (5 emotions × 11 lightness levels). Stimuli were presented in a predetermined fixed order. Within each emotion block, lightness levels were presented in descending order from L0 (100%) to L10 (1%). The order of blocks remained constant across all participants to ensure task consistency and reduce the cognitive load associated with task switching. No practice trials were conducted; participants were allowed to take breaks as necessary. An experimenter was present throughout to monitor procedural adherence and address any questions. This fixed-order design enhanced experimental control, minimized extraneous variability, and supported methodological rigor and reliability.

### 3.4 Results

A series of one-way ANOVAs examined the effect of color lightness on perceived emotional intensity for each of the five emotions. Following APA conventions, we report degrees of freedom, F, and p for each test (Table 1). The omnibus tests were significant for all five emotions—anger (*p* < 0.05), happiness (*p* < 0.01), sadness (*p* < 0.01), fear (*p* < 0.01), and neutral (*p* < 0.01)—indicating that variations in lightness systematically modulated perceived emotional intensity. We refrain from directional claims at this stage; emotion-specific summaries are provided in the subsections below.

**Table 1:** ANOVA results of color lightness.

Intensity of emotions	Color lightness (mean ± standard deviation)											*F*	*p*
	0.0 (*n* = 47)	0.0 (*n* = 47)	0.0 (*n* = 47)	0.0 (*n* = 47)	0.0 (*n* = 47)	0.0 (*n* = 47)	0.0 (*n* = 47)	0.0 (*n* = 47)	0.0 (*n* = 47)	0.0 (*n* = 47)	0.0 (*n* = 47)		
[Anger] changes in the intensity of emotions	2.68 ± 1.32	2.79 ± 1.32	2.98 ± 1.15	3.23 ± 1.03	3.45 ± 0.95	3.40 ± 0.95	3.47 ± 0.83	3.38 ± 0.97	3.21 ± 1.16	3.11 ± 1.62	2.94 ± 1.87	2.31	0.012*
[Sad] changes in the intensity of emotions	2.38 ± 1.28	2.55 ± 1.25	2.68 ± 1.09	3.02 ± 0.90	3.32 ± 0.89	3.60 ± 0.74	3.66 ± 0.73	3.81 ± 0.82	3.62 ± 1.13	3.66 ± 1.40	2.74 ± 1.78	9.99	<0.001
[Neutral] changes in the intensity of emotions	2.55 ± 1.60	2.62 ± 1.41	2.60 ± 1.19	2.8 ± 1.02	3.11 ± 0.98	3.32 ± 0.81	3.34 ± 0.76	3.36 ± 0.76	3.36 ± 1.07	3.17 ± 1.48	2.98 ± 1.80	3.31	<0.001
[Fear] changes in the intensity of emotions	2.19 ± 1.23	2.26 ± 1.09	2.51 ± 1.06	2.72 ± 1.04	2.98 ± 0.92	3.26 ± 1.05	3.45 ± 1.02	3.34 ± 1.01	3.64 ± 1.13	3.70 ± 1.35	3.09 ± 1.73	9.69	<0.001
[Happy] changes in the intensity of emotions	4.70 ± 0.86	4.38 ± 0.80	3.81 ± 0.97	3.40 ± 0.83	3.21 ± 0.86	2.89 ± 0.76	2.60 ± 0.71	2.19 ± 0.65	1.87 ± 0.61	1.45 ± 0.58	1.13 ± 0.54	112	<0.001

*p < 0.05, **p < 0.01.

Figure conventions. Unless stated otherwise, error bars represent SE; sample size is N = 47 for Experiment 1 and *N* = 29 for Experiment 2; lightness mapping follows L0 = 100% … L10 = 1% (higher labels indicate lower lightness); inferential statistics are reported in the corresponding Results subsections.

**Figure 4:**
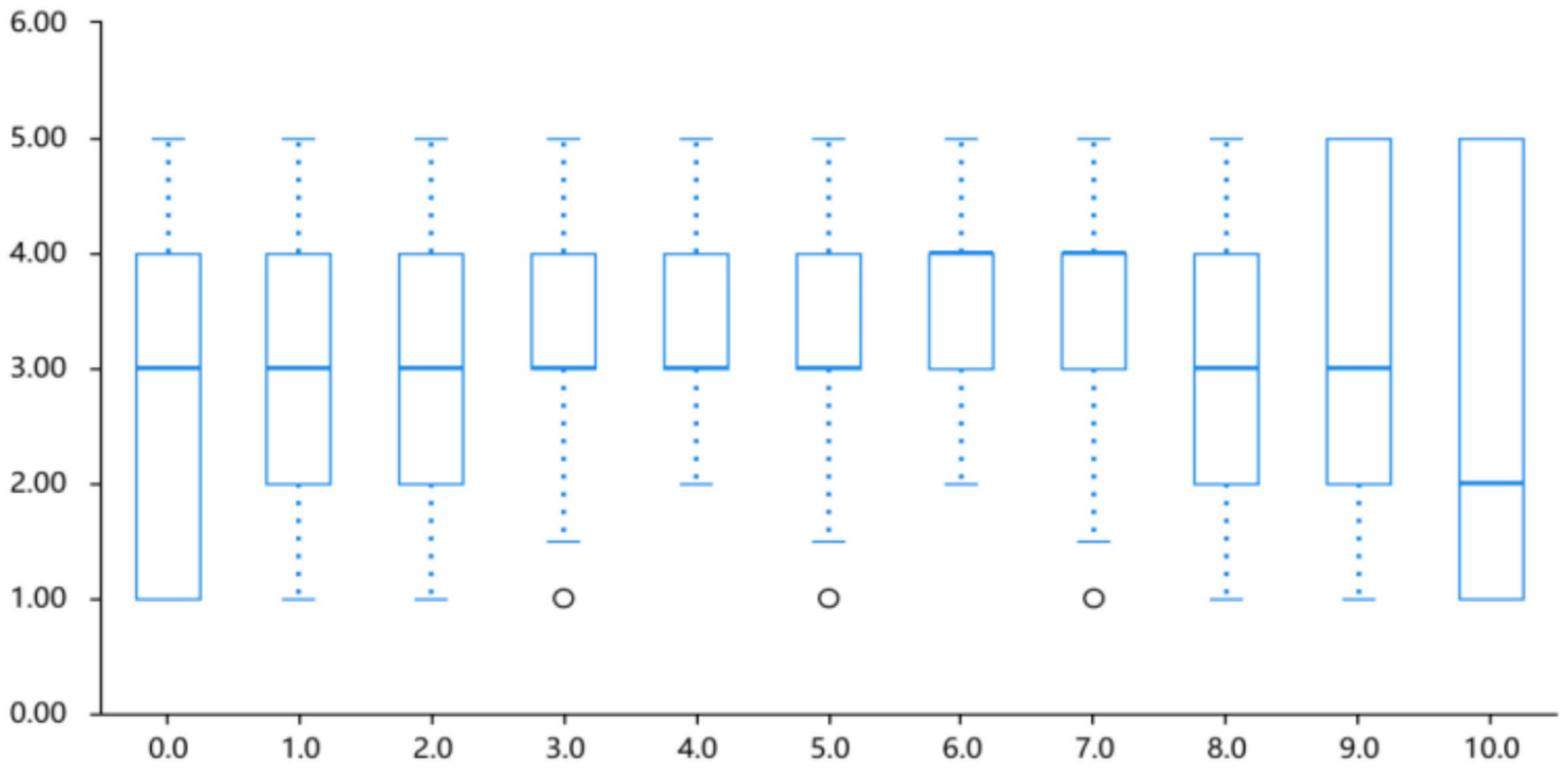
Intensity profile of anger across different lightness conditions.

Anger ratings peaked at lightness levels 6.0–7.0 (40%–30% lightness), with a mean intensity of 3.47 ± 0.83, before declining at darker levels. A simple linear regression showed no significant effect of label (*B* = 0.032, *p* = 0.061; *R*^2^ = 0.007); we refrain from directional claims under the L0 to L10 coding (higher labels = lower lightness). Refer to Table 1 and Figure 4. Linear fits are reported as directional summaries. Where *R*^2^ values are small, inferences are limited, and potential non-linear profiles suggested by visual inspection are flagged as hypotheses for future work rather than claims in the present study.

**Figure 5:**
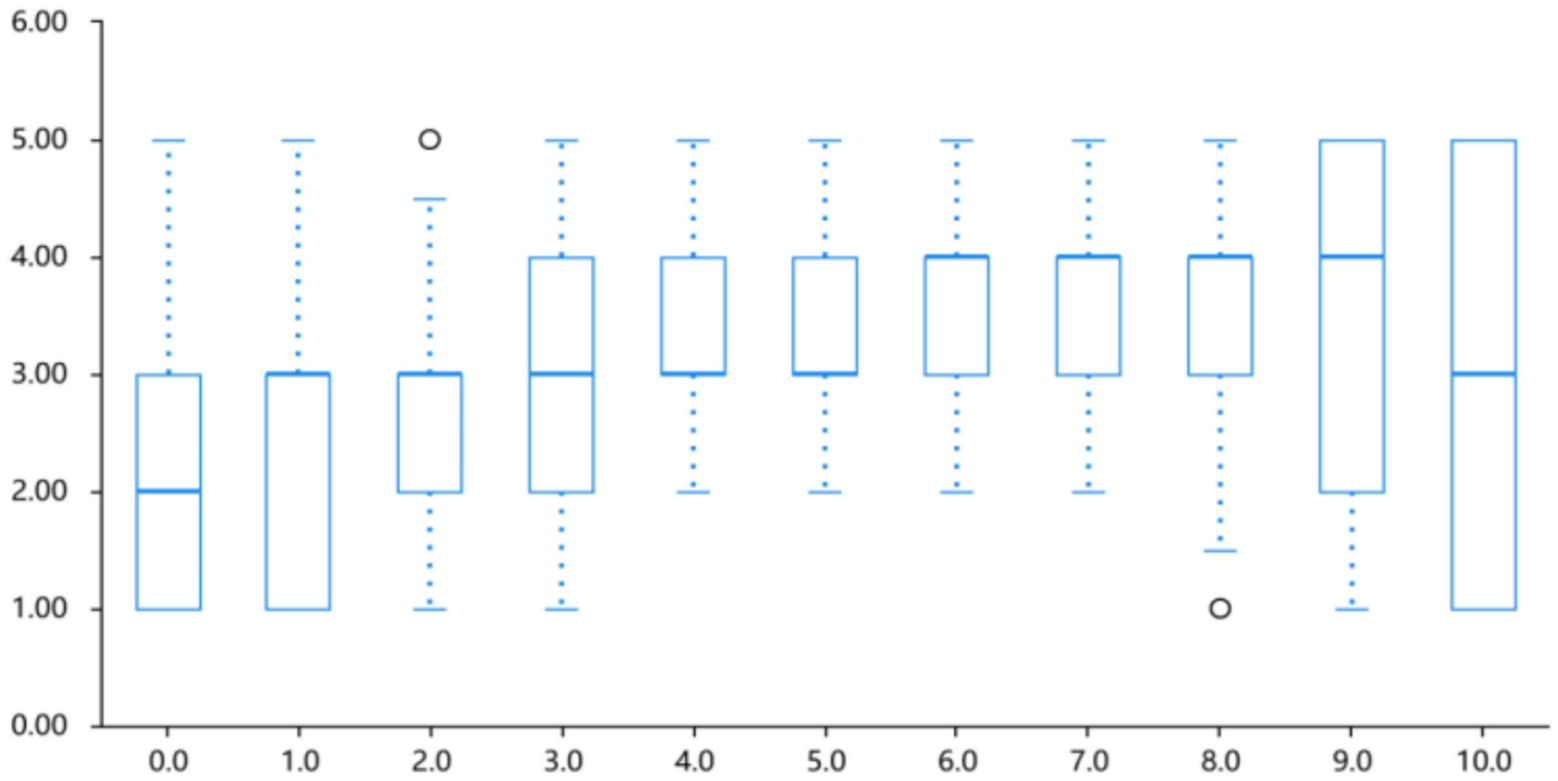
Intensity profile of sadness across different lightness levels.

Sadness ratings peaked at a lightness level of 7.0 (30% lightness, *M* = 3.81 ± 0.82) before declining, with a secondary rise observed at level 9.0 (10% lightness). A simple linear regression revealed a significant linear trend in sadness intensity (*B* = 0.10, *p* < 0.01); however, the explanatory power was modest (*R*^2^ = 0.066). Linear fits are reported as directional summaries; where *R*^2^ values are small, inferences are limited and potential non-linear profiles are noted as hypotheses for future work. Refer to Table 1 and Figure 5.

**Figure 6:**
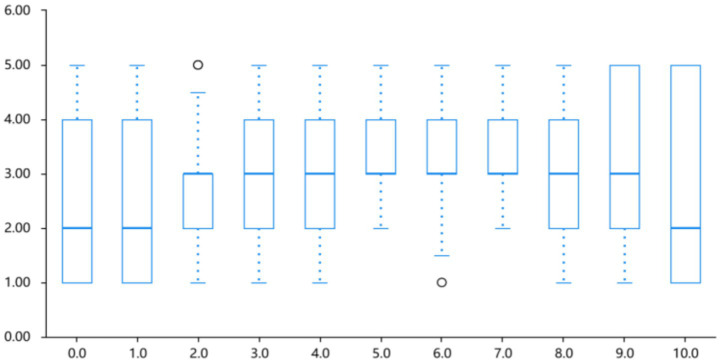
Intensity profile of neutral emotion across different lightness levels.

Neutral expressions peaked at lightness levels 7.0–8.0 (30%–20% lightness; *M* = 3.36 ± 0.76), exhibiting an overall upward trend. A linear regression revealed a significant linear trend in neutral ratings (*B* = 0.072, *p* < 0.01); however, the explanatory power was modest (*R*^2^ = 0.033). We refrain from directional claims given the small *R*^2^ and the L0 to L10 coding (higher labels = lower lightness). Refer to Table 1 and Figure 6.

**Figure 7:**
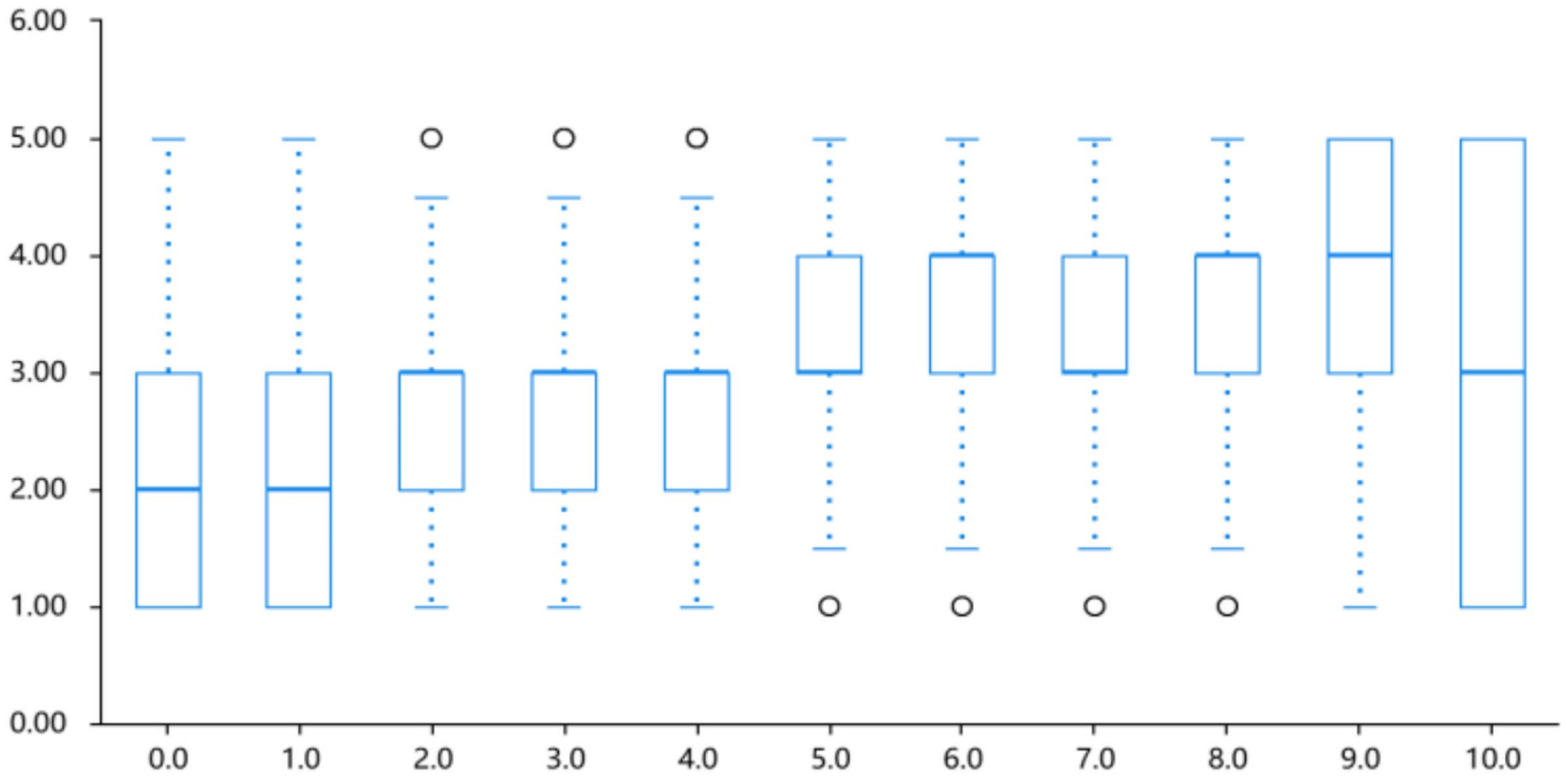
Intensity profile of fear emotion across different lightness levels.

Fear intensity increased significantly as lightness decreased, reaching a peak at lightness level 9.0 (10% lightness; *M* = 3.70 ± 1.35). A linear regression indicated a significant linear trend for fear (*B* = 0.139, *p* < 0.01); the effect size was modest (*R*^2^ = 0.122), so directional interpretations should be made with caution under the L0 to L10 coding. Refer to Table 1 and Figure 7.

**Figure 8:**
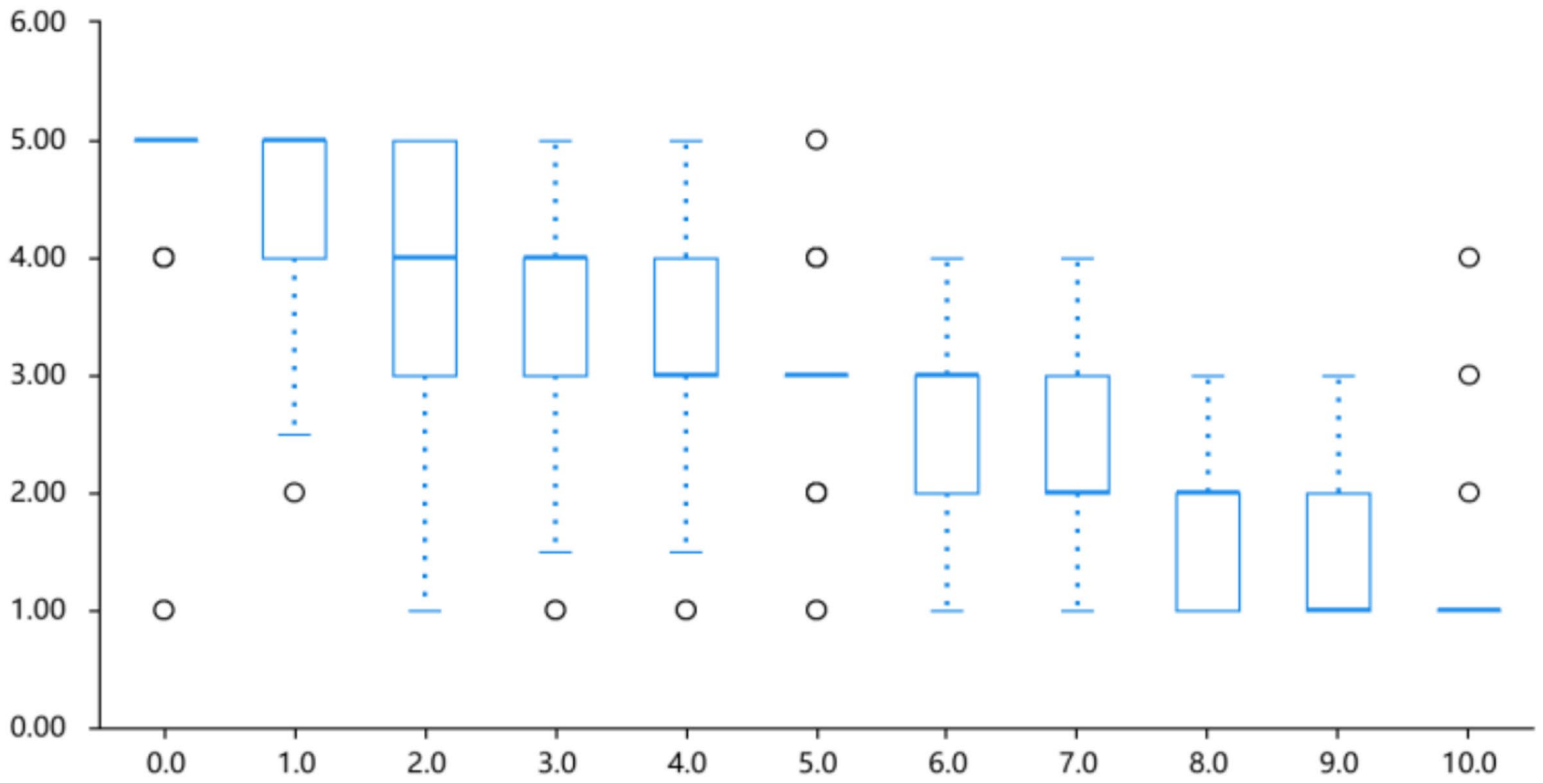
Intensity profile of happy emotion across different lightness levels.

In contrast, happiness intensity decreased significantly as lightness decreased, falling from its maximum at lightness level 0.0 (100% lightness; *M* = 4.70 ± 0.86) to its minimum at level 10.0 (1% lightness; *M* = 1.13 ± 0.54). A linear regression confirmed a significant negative predictive effect of lightness on happiness ratings (*B* = −0.35, *p* < 0.001), indicating that darker colors elicit weaker perceptions of joy. The model exhibited strong explanatory power (*R*^2^ = 0.686), demonstrating that color lightness accounts for a substantial proportion of variance in perceived happiness. Refer to Table 1 and Figure 8.

Overall, color lightness exerts a systematic effect on emotional intensity: darker tones (lower lightness values) amplify negative emotions such as sadness and fear, while significantly diminishing positive emotions like happiness.

## 4 Experiment 2

### 4.1 Materials and methods

In this experiment, an additional group of participants consisting of Chinese undergraduate students was recruited. Consistent with the criteria of Experiment 1, this experiment was an anonymous survey. All participants passed the school - wide standardized physical examination, with normal visual acuity (or corrected - to - normal vision) and normal color perception. The participants had a mean age of 20 years (*N* = 29; 38% female). Face 1 denotes the original timing profile used during data collection, whereas Face 2 denotes a peak-aligned profile motivated by descriptive patterns observed in Experiment 1. This comparison illustrates a design implication (timing alignment) rather than an additional hypothesis test; hence we refrain from further inferential claims here.

### 4.2 Stimuli and apparatus

The stimuli and apparatus employed in this experiment were identical to those in Experiment 1. However, in Experiment 2, a comparative experiment was incorporated. Participants were required to rate dynamic expressions of the same emotion. This comparative experiment involved contrasting the dynamic duration corresponding to the emotional peak identified in Experiment 1 with the original dynamic duration (11 s). For example, in Experiment 1, the time point at which the emotion of “happy” reached its peak arousal level corresponded to the color lightness at the 2nd second. Thus, in the comparative experiment of Experiment 2, the facial expression symbol of “happy” involved dynamic changes in lightness from the 2nd second to the 11th second. Participants were then required to rate each dynamic expression on perceived arousal.

### 4.3 Procedures

The experimental procedures were largely consistent with those of Experiment 1, with the following exceptions: Given the presence of a comparative experiment, participants were tasked with rating two sets of dynamic expressions representing the same emotion but differing in duration. Moreover, since Experiment 2 focused on the dynamic changes in the color lightness of dynamic emoji’s, these were not decomposed into static images.

### 4.4 Results

**Figure 9:**
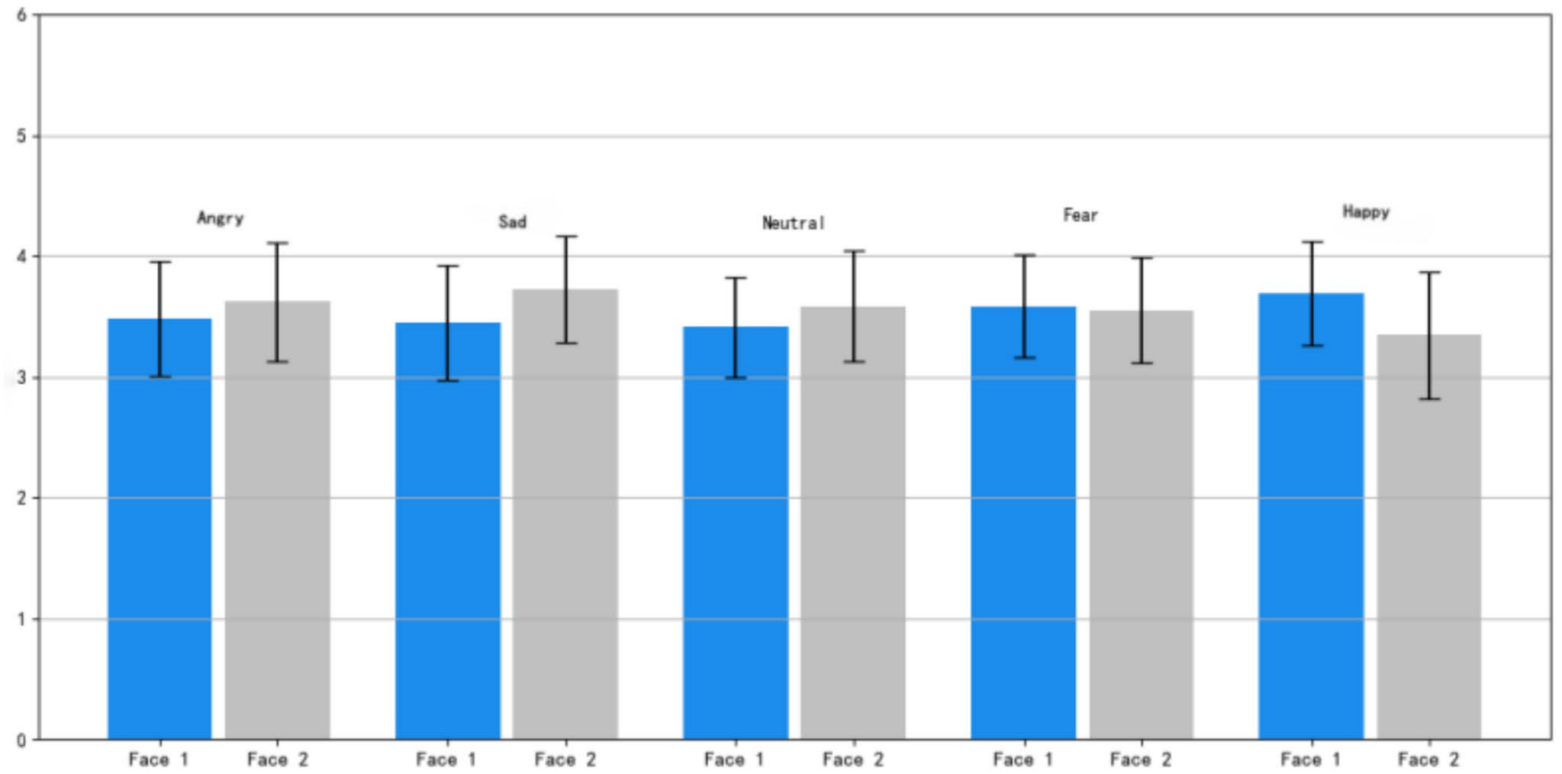
Association between color lightness duration changes and emotional arousal.

Visual inspection of Figure 9 suggests subtle distributional differences between Face 1 and Face 2; the following comparisons are descriptive and not subjected to inferential testing. Analyzing the individual facial emotion feedback, the average ratings of “anger” and “happiness” are marginally higher than those of other emotions. This indicates that the dynamic expressions of these two emotions are more clearly defined and intense in terms of perception. The ratings of “fear” and “neutral” are relatively moderate, whereas the difference in “sadness” is somewhat more pronounced.

Moreover, across all conditions, the error bars (representing standard error) are relatively short. This implies that participants demonstrated good consistency during the evaluation process, and their subjective judgments were relatively stable. Overall, despite minor variances in the dynamic expression of different facial emotions, they generally exhibit a high level of discriminability and consistency.

As can be gleaned from the descriptive data in Table 2 and the rating distributions in Figure 9, when participants viewed Dynamic Expressions 1 and 2, there were certain differences in their perceptions of various emotions. Specifically, the “anger” expression received relatively high and consistent ratings, suggesting that both expressions are effective in conveying the emotion of anger. The “happy” expression ranked second, indicating that it also has good recognizability in expressing positive emotions.

**Table 2:** Emotion ratings associated with dynamic variations in color lightness.

Emotion	Δa* (Face 1)	Δb* (Face 1)	Δa* (Face 2)	Δb* (Face 2)
Anger	10.899484	1.432258	10.899484	1.432258
Sad	−8.662194	−10.238968	−9.328516	−11.026581
Neutral	−5.562000	−9.117161	−5.562000	−9.117161
Fear	5.038710	4.847097	4.992903	4.803032
Happy	1.329	0.997	1.316	0.987

In contrast, the “sad” and “fear” emotions received relatively lower scores in both expressions, and there were certain disparities. This might suggest that the expression of these two types of negative emotions is more nuanced or that there are subjective biases in their recognition. The ratings of the “neutral” emotion were close to the midpoint, conforming to its inherently ambiguous and non-emotional nature.

Overall, dynamic expressions exhibit greater consistency in representing prominent emotions (such as anger and happy). However, when it comes to expressing complex or delicate emotions, which are more latent, they may rely more on individual subjective judgment and comprehension.

## 5 Discussion

### 5.1 The relationship between color lightness variation and emotional arousal in basic emotions

One of the aims of this study is to examine the relationship between the level of emotional arousal (high or low) and the process of color lightness variation. The experimental results indicate that positive emotions (happy) can be elicited without changes in color lightness, whereas negative emotions (sad, fear, and anger) and neutrality require variations in lightness to be aroused. Although previous studies have confirmed that the degree of emotional arousal is related to color variation (Thönes et al., 2018; Ou et al., 2004), the specific lightness thresholds associated with different types of emotions vary. This finding differs from our initial expectation that all emotions would be uniformly associated with color lightness in an inverse manner. These results highlight the necessity of distinguishing among different basic emotions. Specifically, the experiment demonstrates that in robotic facial expressions, happiness does not rely on variations in color lightness; high-lightness colors alone are sufficient to directly convey this emotion. In contrast, emotions such as sadness, fear, anger, and neutrality require changes in color lightness to be effectively elicited.

There are two potential explanations for the finding that changes in color lightness are not associated with the arousal of happy emotions. First, happiness is a type of positive emotion, for which changes in hue and saturation exert a more pronounced influence on emotional arousal. Even when lightness remains constant, positive emotions can be effectively elicited through adjustments in hue and saturation (Jonauskaite et al., 2020; Valdez and Mehrabian, 1994). This may be due to the fact that highly saturated and warm-toned colors (such as yellow) stimulate stronger neural responses in the visual cortex—particularly in areas V4 and V8—and enhance emotional arousal through connections with limbic structures such as the amygdala. Thus, even without changes in lightness, such colors can trigger heightened physiological and subjective arousal (Wilms and Oberfeld, 2018). Second, the semantic associations of colors with everyday life also play a role in emotional elicitation. Specific colors are often closely linked to familiar objects or concepts in the environment, and such semantic connections can evoke positive emotions even in the absence of lightness variations. In fact, even with lower saturation, the intrinsic affective qualities of certain colors can still arouse positive emotional responses (Lin et al., 2024). Therefore, our findings suggest that high-lightness yellow—commonly associated with happiness—can facilitate the arousal of positive emotions without requiring a change in lightness. This is likely due to the combined effects of color-emotion semantics and the inherently uplifting quality of high lightness levels, both of which effectively support the elicitation of positive emotional states.

Sad, fear, anger, and neutral emotions are classified as negative emotions. According to the need for rapid classification of facial expressions in robotics, neutral emotions—due to their lack of positive features and poor recognition validity—are also considered negative emotions (Mollahosseini et al., 2017). The experimental finding that changes in color lightness have an inverse relationship with the arousal of negative emotions can be explained from two perspectives: the physiological mechanisms of color perception and survival-related perceptual responses. Low-lightness colors have been shown to reduce heart rate and skin conductance responses during emotional regulation tasks. Such colors diminish emotional arousal by providing low-lightness stimulation to the visual cortex. Humans also exhibit a protective “withdrawal” mechanism in response to low-lightness environments, thereby suppressing emotional activation (Kaya and Epps, 2004; Vandewalle et al., 2009; Elliot and Maier, 2014). In visual psychology, low-lightness colors are commonly associated with “suppressive” or “calming” effects, as they reduce excitability within the emotional system—particularly for emotions such as fear and sadness. Additionally, the expression of negative emotions is shaped by color associations grounded in environmental and cultural experience. In many cultures, low-lightness colors such as black, gray, and dark blue are symbolically tied to emotions like sadness and grief. Over time, these culturally embedded associations lead to desensitization or habituation, weakening the arousal potency of such colors (Cajochen, 2007; Ou et al., 2012; Gao et al., 2007). Therefore, it can be inferred that the emotional regulation of negative states may be more precisely achieved through transitions from high-lightness to low-lightness colors. This transformation aligns with findings in neuroscience, where low-lightness visual stimuli affect brain regions involved in emotion, such as the amygdala and lateral habenula. These stimuli activate the parasympathetic nervous system, enhance alpha wave activity, and reduce heart rate—thereby inducing and modulating negative emotional states (Li et al., 2021; Park et al., 2013). Furthermore, the long-term cultural conditioning that associates low-lightness colors with loss and sadness further reinforces their emotion-suppressing function, which should be taken into account in interpreting their role in emotional arousal. In our data, lower lightness was associated with higher perceived intensity for several negative emotions (e.g., fear and sadness; see Section 3.4). Effects likely depend on emotion category and display context, and may be non-linear.

### 5.2 The color lightness of different basic emotions is correlated with peaks in emotional arousal

The second objective was to compare how the color lightness associated with different basic emotions influences emotional arousal. In the experiment, we observed that when using the default lightness values of Plutchik’s Emotion Wheel, anger, sadness, and neutral emotions reached peak arousal at a lightness level of 30%, whereas fear and sadness peaked at a lower lightness of 10%. Interestingly, sadness elicited arousal peaks at both 10% and 30% lightness, suggesting a dual sensitivity to both moderate and low lightness levels. This pattern supports the idea that color lightness and saturation independently contribute to emotional arousal and aligns with previous findings that decreased lightness (i.e., lower lightness) is often linked with heightened arousal in negative emotional states (Fugate and Franco, 2019). Notably, the lightness values of negative emotions—such as anger, sadness, and even neutral emotions—do not necessarily reach arousal peaks at the lowest lightness. Rather, darker colors (low lightness) tend to elicit high-alert physiological responses (e.g., fear), whereas medium lightness more readily evokes high-energy emotions like anger (Sutton and Altarriba, 2016; Weng et al., 2024). From this perspective, a lightness value of 30% appears optimal for conveying facial expressions of anger, sadness, and neutral emotions, while an extremely low lightness of 10% may be more effective for fear and intensified sadness. The dual-peak response of sadness—at both 10% and 30%—also reflects the inverted-U model of the Yerkes–Dodson law, where optimal arousal can occur at moderate levels of stimulation, but secondary arousal peaks may emerge at extreme ends due to emotional intensification (Yerkes and Dodson, 1908; Diamond, 2005; Koob and Le Moal, 2008). For anger and neutral emotional states, a 30% lightness level offers a visually processable, moderate-intensity color stimulus, often associated with a “melancholic” or “nostalgic” type of sadness. In contrast, extremely dark colors (10% lightness) may trigger “deep sorrow” or “intense suppression,” leading to perceptual disfluency and consequently, a stronger sense of alertness or heaviness (Reber et al., 2004; Forster, 2020; Gray et al., 2011). On the other hand, happiness reached its arousal peak at the highest lightness level (100%), a result consistent with the positive correlation between lightness and emotional arousal in the model proposed by Valdez and Mehrabian (1994). The steepest arousal slope occurred at maximum lightness, aligning with the “optimal performance–maximum arousal” model described by the Yerkes–Dodson law (Wilms and Oberfeld, 2018). Moreover, in grouped design conditions, participants reported significantly higher aesthetic and emotional activation ratings under high-lightness stimuli, compared to medium or low-lightness conditions (Shen et al., 2010).

Therefore, in this study, the robot’s facial emotion expression process revealed significant differentiation among various emotion types during the color lightness-induced arousal process. Notably, the emotion of sadness exhibited a bimodal effect, aligning with the inverted U-shaped arousal pattern as well as the nonlinear modulation between processing fluency and cognitive congruence. To achieve accurate emotional transmission, the robot’s facial design should dynamically adjust color lightness based on the specific emotion type, thereby balancing perceptual fluency with emotional alertness and ensuring the effective expression of both negative and positive emotions (Küller et al., 2006; Chebat and Morrin, 2007). In the context of practical human–robot interaction applications, environmental perception mapping (e.g., adjusting the target lightness in a brighter room to maintain the contrast between the human face and the background) and straightforward calibration cues can enhance the stability of perception across diverse devices and environments. These recommendations do not aim to instantaneously alter the established patterns. Instead, they serve to clarify how such patterns can be effectively translated into practical applications in the future.

### 5.3 The correlation between the duration of facial color lightness changes and emotion transmission

The third objective of this study was to explore the relationship between the dynamic variations in color lightness within robotic facial expressions and emotions. During the experiment, it was observed that the dynamic changes in color lightness demonstrated greater consistency when conveying prominent emotions such as anger and happiness. Conversely, the expression of complex or delicate latent emotions seemed to rely more heavily on individual subjective judgment and comprehension. There exists a profound inherent connection between the duration of facial color lightness changes and emotion transmission. From the perspective of the fundamental logic of emotion transmission, the temporal dimension of lightness changes, serving as a crucial visual cue in dynamic expressions, effectively mimics the basic physiological and social emotional information transmitted by humans in normal circumstances (Jack et al., 2014). When communicating prominent emotions like anger and happiness, the relatively stable and regular duration of lightness changes aligns with the human cognitive expectation of “decisiveness and directness” for intense emotions (Fayolle and Droit, 2014). For example, in the process of identifying others’ emotions through facial expressions, the external manifestations of intense emotions typically exhibit a clear and distinct rhythm. This visual rhythm, transmitted through the dynamic changes in color lightness, can rapidly activate the corresponding emotion cognitive modules, thereby strengthening the certainty of emotion recognition. In contrast, when dealing with complex emotions such as sadness and fear, the subtle differences and uncertainties in the duration of lightness changes reveal the ambiguous and multi-faceted nature of human complex emotions (Recio et al., 2013). In real-life scenarios, latent emotions are more vulnerable to the influence of individual experiences and cognitive biases. This also makes the brain more sensitive to the subtle variations in the visual rhythm of emotions. As a result, during the process of emotion transmission, it is challenging to precisely generalize with a single, fixed visual rhythm, thus providing more space for subjective emotional interpretation (Pérez-Edgar et al., 2014). Therefore, the transmission of latent emotions through facial lightness changes is more reliant on individual subjective cognition and gives rise to more uncertainties. On the other hand, the characteristics of lightness changes in neutral emotions are more congruent with the essence of “de-emotionalization.” By maintaining a gentle and stable visual rhythm, they uphold the neutrality of emotion expression and avoid strong emotional biases (Suess et al., 2015). From the perspectives of emotional communication and social significance, the regulation of emotion transmission by the duration of lightness changes essentially constructs a set of visual “emotional language” rules. The highly consistent transmission pattern of overt emotions is conducive to quickly reaching emotional consensus, meeting the requirements for rapid responses to crucial emotions in survival and social interactions. The ambiguous transmission pattern of latent emotions, although increasing the difficulty of recognition, reserves flexible space for emotional communication.

Prior to the experiment, participants were explicitly informed that the same emotion would be presented under different levels of lightness. This practice was conducive to ensuring the understanding of the task. However, it might have exacerbated the expectancy effect. In subsequent research, the impact of demand characteristics will be mitigated through means such as more implicit instructions, between—subject’s designs, or masking tasks. This study adopted a fixed order of decreasing lightness (L0 to L10) to ensure score consistency and reduce the burden of task switching. This approach is easy to understand and operate, but it may bring about an expectation or contrast effect. Therefore, when R^2^ is relatively small, we avoid emphasizing the directional conclusions of the research and regard possible nonlinearity as assumptions. Subsequent studies will adopt a more balanced or random sequence to reduce sequence confusion.

## 6 Conclusion

This research undertook an in-depth investigation into the relationship between diverse emotion types and color lightness within the facial expression symbols of social robots. The static analysis in Experiment 1 and the dynamic validation in Experiment 2 can more precisely reveal the relationship between the dynamic changes in the color lightness of the facial expression symbols of social robots and emotional arousal. An experiment was carried out, categorizing facial expressions according to the emotion colors in Plutchik’s Emotion Wheel and the lightness values in the Munsell color system. The study ultimately found that positive emotions can be directly evoked by colors of high lightness. In contrast, negative emotions and neutral emotions necessitate a process of decreasing lightness to intensify emotional arousal. Across emotions, lower lightness tended to amplify the perceived intensity of several negative emotions (e.g., sadness, fear) while reducing perceived happiness; effects appear emotion-specific and may be non-linear. Optimal lightness values vary across different emotions. Happiness attains its arousal peak at high lightness levels. Emotions such as anger and sadness reach their peaks at approximately 30% lightness, and sadness may exhibit a secondary peak at extremely low lightness, following an inverted-U pattern. Furthermore, the dynamic changes in color lightness demonstrate greater consistency in the expression of prominent emotions (e.g., anger, happy). Emotions can be accurately conveyed through brief durations of change. Conversely, when expressing complex or delicate latent emotions (e.g., sad, fear), the process may be more reliant on individual subjective judgment and comprehension.

## Data Availability

The original contributions presented in the study are included in the article/supplementary material, further inquiries can be directed to the corresponding author.

## References

[ref2] Bosten J. M. (2022). Do you see what I see? Diversity in human color perception. Annu. Rev. Vis. Sci. 8, 101–133. doi: 10.1146/annurev-vision-093020-112820 35709507

[ref4] Cai Y. Li X. Li J. (2023). Emotion recognition using different sensors, emotion models, methods and datasets: a comprehensive review. Sensors 23, 2455–2488. doi: 10.3390/s23052455 36904659 PMC10007272

[ref5] Cajochen C. (2007). Alerting effects of light. Sleep Med. Rev. 11, 453–464. doi: 10.1016/j.smrv.2007.07.009, 17936041

[ref7] Casimir M. J. Schnegg M. (2002). “Shame across cultures: the evolution, ontogeny and function of a ‘moral emotion’” in Between culture and biology: perspectives on ontogenetic development. eds. Keller H. Poortinga Y. H. Schölmerich A. (Cambridge: Cambridge University Press).

[ref8] Chebat J. C. Morrin M. (2007). Colors and cultures: exploring the effects of mall décor on consumer perceptions. J. Bus. Res. 60, 189–196. doi: 10.1016/j.jbusres.2006.11.003

[ref9] Chuah S. H. W. Yu J. (2021). The future of service: the power of emotion in human-robot interaction. J. Retail. Consum. Serv. 61, 2551–2559. doi: 10.1016/j.jretconser.2021.102551

[ref10] Corney D. Haynes J. D. Rees G. Lotto R. B. (2009). The brightness of colour. PloS One 4:e5091. doi: 10.1371/journal.pone.0005091, 19333398 PMC2659800

[ref12] Diamond D. M. (2005). Cognitive, endocrine and mechanistic perspectives on non-linear relationships between arousal and brain function. Nonlinearity Biol. Toxicol. Med. 3, 1–7. doi: 10.2201/nonlin.003.01.001, 19330153 PMC2657838

[ref16] Elliot A. J. Maier M. A. (2014). Color psychology: effects of perceiving color on psychological functioning in humans. Annu. Rev. Psychol. 65, 95–120. doi: 10.1146/annurev-psych-010213-115035 23808916

[ref9003] Fayolle S. L. Droit-Volet S. (2014). Time perception and dynamics of facial expressions of emotions. PLOS ONE, 9:e97944. 24835285 10.1371/journal.pone.0097944 PMC4023999

[ref17] Forster M. (2020). The Oxford handbook of empirical aesthetics. Oxford: Oxford University Press.

[ref18] Fugate J. M. B. Franco C. L. (2019). What color is your anger? Assessing color-emotion pairings in English speakers. Front. Psychol. 10:206. doi: 10.3389/fpsyg.2019.00206 30863330 PMC6399154

[ref19] Gao X. P. Xin J. H. Sato T. Hansuebsai A. Scalzo M. Kajiwara K. . (2007). Analysis of cross-cultural color emotion. Color Res. Appl 32, 223–229. doi: 10.1002/col.20321

[ref20] Giard F. Guitton M. J. (2010). Beauty or realism: the dimensions of skin from cognitive sciences to computer graphics. Comput. Hum. Behav. 26, 1748–1752. doi: 10.1016/j.chb.2010.07.001

[ref21] Gray H. M. Ishii K. Ambady N. (2011). Misery loves company: when sadness increases the desire for social connectedness. Personal. Soc. Psychol. Bull. 37, 1438–1448. doi: 10.1177/0146167211420167, 21873533

[ref23] Jack R. E. Garrod O. G. Schyns P. G. (2014). Dynamic facial expressions of emotion transmit an evolving hierarchy of signals over time. Curr. Biol. 24, 187–192. doi: 10.1016/j.cub.2013.11.064, 24388852

[ref9002] Izard C. E. (2007). Basic emotions, natural kinds, emotion schemas, and a new paradigm. Perspectives on Psychological Science, 2, 260–280. 26151969 10.1111/j.1745-6916.2007.00044.x

[ref24] Jonauskaite D. Abu-Akel A. Dael N. Oberfeld D. Abdel-Khalek A. M. Al-Rasheed A. S. . (2020). Universal patterns in color-emotion associations are further shaped by linguistic and geographic proximity. Psychol. Sci. 31, 1245–1260. doi: 10.1177/0956797620948810, 32900287

[ref25] Jonauskaite D. Althaus B. Dael N. Dan-Glauser E. Mohr C. (2019). What color do you feel? Color choices are driven by mood. Color Res. Appl. 44, 272–284. doi: 10.1002/col.22327

[ref26] Kaya N. Epps H. H. (2004). Relationship between color and emotion: a study of college students. Coll. Stud. J. 38, 396–405.

[ref27] Koob G. F. Le Moal M. (2008). Neurobiological mechanisms for opponent motivational processes in addiction. Philos. Trans. R. Soc. Lond. B Biol. Sci. 363, 3113–3123. doi: 10.1098/rstb.2008.0094 18653439 PMC2607326

[ref28] Küller R. Ballal S. Laike T. Mikellides B. Tonello G. (2006). The impact of light and colour on psychological mood: a cross-cultural study of indoor work environments. Ergonomics 49, 1496–1507. doi: 10.1080/00140130600858142, 17050390

[ref29] Kuniecki M. Pilarczyk J. Wichary S. (2015). The color red attracts attention in an emotional context. An ERP study. Front. Hum. Neurosci. 9:212. doi: 10.3389/fnhum.2015.00212 25972797 PMC4413730

[ref30] Lapate R. C. Rokers B. Li T. Davidson R. J. (2014). Nonconscious emotional activation colors first impressions: a regulatory role for conscious awareness. Psychol. Sci. 25, 349–357. doi: 10.1177/0956797613503175, 24317420 PMC4070508

[ref31] Li Y. Ru T. Chen Q. Qian L. Luo X. Zhou G. (2021). Effects of illuminance and correlated color temperature of indoor light on emotion perception. Sci. Rep. 11:14351. doi: 10.1038/s41598-021-93523-y 34253773 PMC8275593

[ref32] Liao S. Sakata K. Paramei G. V. (2018). “Color affects the intensity of emotions read out from emoticons.” In: The Proceedings of the Annual Convention of the Japanese Psychological Association. Sendai: Sendai International Center

[ref33] Liao S. Sakata K. Paramei G. V. (2022). Color affects recognition of emoticon expressions. Iperception 13, 1–23. doi: 10.1177/20416695221080778 PMC8900290 35265312

[ref35] Lin C. Mottaghi S. Shams L. (2024). The effects of color and saturation on the enjoyment of real-life images. Psychon. Bull. Rev. 31, 361–372. doi: 10.3758/s13423-023-02357-4, 37620633 PMC10867063

[ref37] Luo Q. Dzhelyova M. (2020). Consistent behavioral and electrophysiological evidence for rapid perceptual discrimination among the six human basic facial expressions. Cogn. Affect. Behav. Neurosci. 20, 928–948. doi: 10.3758/s13415-020-00811-7, 32918269

[ref38] Mavridis N. (2015). A review of verbal and non-verbal human–robot interactive communication. Robot. Auton. Syst. 63, 22–35. doi: 10.1016/j.robot.2014.09.031

[ref40] Mollahosseini A. Hasani B. Mahoor M. H. (2017). AffectNet: a database for facial expression, valence, and arousal computing in the wild. IEEE Trans. Affect. Comput. 10, 18–31. doi: 10.1109/TAFFC.2017.2740923

[ref41] Mourao-Miranda J. Volchan E. Moll J. de Oliveira-Souza R. Oliveira L. Bramati I. . (2003). Contributions of stimulus valence and arousal to visual activation during emotional perception. NeuroImage 20, 1955–1963. doi: 10.1016/j.neuroimage.2003.08.011, 14683701

[ref42] Nakajima K. Minami T. Nakauchi S. (2017). Interaction between facial expression and color. Sci. Rep. 7:41019. doi: 10.1038/srep41019 28117349 PMC5259783

[ref44] Ou L. C. Luo M. R. Woodcock A. Wright A. (2004). A study of colour emotion and colour preference. Part 1: colour emotions for single colours. Color Res. Appl. 29, 232–240. doi: 10.1002/col.20010

[ref45] Ou L. C. Ronnier Luo M. Sun P. L. Hu N. C. Chen H. S. Guan S. S. . (2012). A cross-cultural comparison of colour emotion for two-colour combinations. Color. Res. Appl. 37, 23–43. doi: 10.1002/col.20648

[ref47] Park J. Y. Ha R. Y. Ryu V. Kim E. Jung Y. C. (2013). Effects of color temperature and lightness on electroencephalogram alpha activity in a polychromatic light-emitting diode. Clin. Psychopharmacol. Neurosci. 11, 126–131. doi: 10.9758/cpn.2013.11.3.126, 24465248 PMC3897760

[ref48] Pérez-Edgar K. Hardee J. E. Guyer A. E. Benson B. E. Nelson E. E. Gorodetsky E. . (2014). DRD4 and striatal modulation of the link between childhood behavioral inhibition and adolescent anxiety. Soc. Cogn. Affect Neurosci. 9, 445–453. doi: 10.1093/scan/nst001 23314010 PMC3989122

[ref49] Peromaa T. Olkkonen M. (2019). Red color facilitates the detection of facial anger—But how much? PLoS One 14:e0215610. doi: 10.1371/journal.pone.0215610, 30995286 PMC6469786

[ref9001] Plutchik R. (1980). Emotion: A psychoevolutionary synthesis. Harper & Row.

[ref50] Reber R. Schwarz N. Winkielman P. (2004). Processing fluency and aesthetic pleasure: Is beauty in the perceiver’s processing experience? Personal. Soc. Psychol. Rev. 8, 364–382. doi: 10.1207/s15327957pspr0804_3, 15582859

[ref51] Recio G. Schacht A. Sommer W. (2013). Classification of dynamic facial expressions of emotion presented briefly. Cognit. Emot. 27, 1486–1494. doi: 10.1080/02699931.2013.794128, 23659578

[ref53] Riordan M. A. (2017). Emojis as tools for emotion work: communicating affect in text messages. J. Lang. Soc. Psychol. 36, 549–567. doi: 10.1177/0261927x17704238

[ref54] Russell J. A. (1980). A circumplex model of affect. J. Pers. Soc. Psychol. 39, 1161–1178. doi: 10.1037/h0077714

[ref56] Shen H. Jiang Y. Adaval R. (2010). Contrast and assimilation effects of processing fluency. J. Consum. Res. 36, 876–889. doi: 10.1086/612425

[ref57] Spezialetti M. Placidi G. Rossi S. (2020). Emotion recognition for human-robot interaction: recent advances and future perspectives. Front. Robot. AI 7, 79–89. doi: 10.3389/frobt.2020.532279 33501307 PMC7806093

[ref59] Suess F. Rabovsky M. Abdel Rahman R. (2015). Perceiving emotions in neutral faces: expression processing is biased by affective person knowledge. Soc. Cogn. Affect. Neurosci. 10, 531–536. doi: 10.1093/scan/nsu088, 24948155 PMC4381241

[ref61] Sutton T. M. Altarriba J. (2016). Color associations to emotion and emotion-laden words: a collection of norms for stimulus construction and selection. Behav. Res. Methods 48, 686–728. doi: 10.3758/s13428-015-0598-8, 25987304

[ref63] Thanh Huyen D. Nghia Mai T. Tanaka S. Yamada K. (2025). Color-based models for emotions expressing for robosts: a Vietnamese pilot research. Vietnam J. Comput. Sci., 1–15. doi: 10.1142/S2196888825500149

[ref64] Thönes S. Von Castell C. Iflinger J. Oberfeld D. (2018). Color and time perception: evidence for temporal overestimation of blue stimuli. Sci. Rep. 8, 1688–1696. doi: 10.1038/s41598-018-19892-z, 29374198 PMC5786107

[ref65] Valdez P. Mehrabian A. (1994). Effects of color on emotions. J. Exp. Psychol. Gen. 123, 394–409. doi: 10.1037/0096-3445.123.4.394, 7996122

[ref66] Van Kleef G. A. Côté S. (2022). The social effects of emotions. Annu. Rev. Psychol. 73, 629–658. doi: 10.1146/annurev-psych-020821-010855 34280326

[ref67] Vandewalle G. Maquet P. Dijk D. J. (2009). Light as a modulator of cognitive brain function. Trends Cogn. Sci. 13, 429–438. doi: 10.1016/j.tics.2009.07.004, 19748817

[ref68] Weng H. C. Julvanichpong T. Jaidee P. Piboon K. Inchai P. Imcha L. . (2024). Emotional expression and mental health: decoding color and drawing styles with Python and OpenCV. Asian J. Soc. Health Behav. 7, 116–122. doi: 10.4103/shb.shb

[ref69] Wilms L. Oberfeld D. (2018). Color and emotion: effects of hue, saturation, and lightness. Psychol. Res. 82, 896–914. doi: 10.1007/s00426-017-0880-8, 28612080

[ref70] Yerkes R. M. Dodson J. D. (1908). The relation of strength of stimulus to rapidity of habit-formation. J. Comp. Neurol. Psychol. 18, 459–482. doi: 10.1002/cne.920180503

